# Distinctive Features of Rotational Thromboelastometry in Patients with Infective Endocarditis Undergoing on-Pump Cardiac Surgery: A Retrospective Propensity Score-matched Observational Study

**DOI:** 10.1177/10760296251356205

**Published:** 2025-07-03

**Authors:** Stanislaw Vander Zwaag, Imre Kukel, Kinga Towarek-Nocon, Jens Fassl

**Affiliations:** 1 Department of Cardiac Anesthesiology, Medical Faculty and Heart Center Dresden, 39074 TUD Dresden University of Technology, Dresden, Germany; 2 Department of Anaesthesiology, Intensive Care, Emergency-, Pain- and Palliative Medicine, 39492 Elblandklinikum Radebeul, Radebeul, Germany

**Keywords:** endocarditis, coagulation, viscoelastic tests, ROTEM, patient blood management

## Abstract

**Background:**

The haemostatic system plays a role in development of valvular vegetations and systemic activation of coagulation, contributing to both thrombosis and consumption coagulopathy. Patients with infective endocarditis undergoing valvular surgery pose unique challenges to the surgical team. Rotational thromboelastometry (ROTEM), a point-of-care viscoelastic clotting test, can help assess coagulation and guide therapy. This retrospective propensity score-matched study explores ROTEM's distinctive features in endocarditis patients compared to others undergoing cardiac surgery.

**Materials and Methods:**

A retrospective analysis was performed on patients undergoing valvular surgery between May 31, 2022, and August 31, 2024. ROTEM assessments were conducted during extracorporeal circulation and post-heparin reversal. The study group consisted of patients with endocarditis, while propensity score matching created a control group of patients without endocarditis. The primary outcome was on-pump ROTEM results; secondary outcomes included utilisation of red blood cells (pRBC), fibrinogen concentrate (FC), prothrombin complex concentrate (PCC), fresh frozen plasma (FFP), and platelets.

**Results:**

In both groups (23 patients each), endocarditis patients had prolonged EXTEM clotting times (136 vs 96 s, *P* = .001) and higher FIBTEM-A5 clot firmness (19 vs 13 mm, *P* = .012). They required more PCC (3000 vs 2500 units, *P* = .014), while transfusions of platelets (*P* = .281), FFP (*P* = 1.000), and FC (*P* = .109) did not differ significantly.

**Conclusion:**

Endocarditis patients demonstrate distinct viscoelastic profiles with prolonged clotting times and increased clot firmness, necessitating greater PCC supplementation. Further research is warranted to optimise ROTEM target values to balance bleeding and thrombotic risks.

## Introduction

It is well established that the haemostatic system plays a pivotal role in the pathogenesis of infective endocarditis (IE).^1,2^ Following bacteraemia, the formation of endocardial vegetations is a potentially dangerous consequence. These structures, attached to one or more of the cardiac valves, consist of the core of an infected thrombus covered with fibrin and platelets. This coating protects the bacteria from the immune system.^1,2^

The presence of a bloodstream infection that results in IE gives rise to a complex response in the coagulation system, a phenomenon referred to as immunothrombosis.^3^ The release of cytokines from endothelial cells, leukocytes, and platelets after recognition of bacteria results in endothelial activation. The release of tissue factor from the endothelium and leukocytes results in a shift from an anticoagulant to a prothrombotic state due to the activation of the extrinsic pathway. Concurrently, the intrinsic system is triggered by the DNA or RNA of dying cells as well as by bacterial cell wall components.^4^

Thrombin generated in this process plays a role in inflammation via the thrombomodulin-protein C pathway and thrombin receptors on platelets, endothelial cells, and leukocytes.^3^

Although the aforementioned system is robustly balanced under normal circumstances, fulminant infections may disrupt it, leading to bleeding or thrombosis. In the case of IE, an attempt to contain the infection results in providing the pathogen with an optimal environment to thrive.^2^

IE is associated with a high mortality rate largely due to local and systemic complications. Despite the administration of optimal therapy, mortality rates remain as high as twenty-four percent.^5^ Destruction of cardiac valves, valvular regurgitation, rapid progression of heart failure, and septic emboli contribute to this unfavourable outcome.^2,6^ Furthermore, intracranial haemorrhage may occur in 7%-27% of patients with IE, with microhaemorrhagic lesions reported in up to 57%.^7–10^ Therefore, any treatment with anticoagulant, antithrombotic, or antiplatelet drugs aimed at interfering with the formation of vegetation carries an inherent risk of intracranial bleeding.

Approximately 50% of IE patients undergo cardiac surgery at tertiary care centers.^11^ The perioperative management of these patients presents a number of unique challenges, including the potential for both bleeding and thrombotic complications. The use of rotational thromboelastometry (ROTEM) as a point-of-care examination has the potential to facilitate individualization of management strategies.

In ROTEM, citrate blood samples are analyzed to evaluate coagulation. In particular, INTEM initiates the coagulation cascade through contact with the sample and activation of the intrinsic pathway, whereas EXTEM uses tissue factor to trigger the extrinsic pathway. The addition of heparinase to the INTEM assay permits the exclusion of the heparin effect in HEPTEM. In FIBTEM, cytochalasin D is employed to inhibit the function of thrombocytes, thereby enabling the assessment of the isolated fibrinogen contribution to clot firmness.^12^

The objective of the present retrospective observational propensity score-matched study was to examine the distinctive features of ROTEM results in patients with IE compared to the general population undergoing cardiac surgery.

## Methods

This study was conducted in accordance with the Declaration of Helsinki and its amendments. Ethical approval was granted by the Ethics Committee of the Medical Faculty Carl Gustav Carus of the TUD Dresden University of Technology (Chairperson: Prof. B. Renner) on August 12, 2024 (Decision # BO-EK-273072024). Owing to the retrospective design of the study, the requirement for informed consent was waived in accordance with German legislation.

### Setting and Participants

This study was conducted at a single academic cardiac centre. A database of ROTEM examinations conducted between 31st of May 2022 and 31st of August 2024 was screened to identify adult patients with infective endocarditis who underwent two examinations during valve surgery: one during the end phase of cardiopulmonary bypass (CPB) and one after protamine administration. The first time point was chosen because it was predictive of post-heparin reversal values, allowing for better preparation and workflow after weaning from CPB.^13^ Patients with hereditary bleeding disorders and those on direct oral anticoagulants, which could not be paused for the recommended time preoperatively, were excluded from the study. Patients undergoing cardiac surgery without infective endocarditis who met the same criteria were subjected to propensity score matching to form a control group. Owing to the retrospective design of this study, the study sample was not calculated; instead, all data available in the ROTEM device database were screened for eligible patients.

### Coagulation Management

One gram of tranexamic acid was administered after induction of anaesthesia, according to the institutional protocol. The patients did not receive any additional supplementation prior to the on-pump ROTEM examination. To permit the initiation of extracorporeal circulation, 500 IU of heparin per kilogram (rounded up to 5000 IU up to max. 50 000 IU) was administered. After reversal with protamine (1 mg to 1000 IU initial heparin), one gram of tranexamic acid was repeated, and human fibrinogen concentrate (FC), 4-factor prothrombin complex concentrate (PCC), fresh frozen plasma (FFP), and platelets were administered based on ROTEM results, and clinical microvascular bleeding was observed.

The institutional protocol for ROTEM-based coagulation management involves carrying out a routine ROTEM test at the end of cardiopulmonary bypass for patients at high risk of bleeding (including those with IE). Based on the results, clinicians may administer fibrinogen concentrate, PCC or platelets as first-line therapy. At the time, the dose of these products was not standardised and was at the discretion of the anaesthetist treating the patient. Anaesthesiologists were advised to administer fibrinogen concentrate first if A5 was low in FIBTEM and CT was prolonged in EXTEM. However, they were permitted to administer PCC and FC simultaneously in cases of severely prolonged CT in EXTEM with reasonable A5 in FIBTEM. Plasma was only administered to patients with severely prolonged CT in both EXTEM and INTEM.

### Surgical Management

All patients were operated on by one of eight senior cardiac surgeons, each with a similar level of expertise. Minimally invasive surgical techniques via lateral access were performed wherever feasible. Seven perfusionists operated the cardiopulmonary bypass following the same institutional protocol. The same surgeons and perfusionists operated the patients in both investigated groups.

### Blood Sampling

A total of 6 ml of blood was sampled using citrate tubes. During extracorporeal circulation, blood was drawn from the arterial line of the cardiopulmonary bypass. Following the administration of protamine, further samples were taken from the radial arterial line.

### Outcomes

The primary outcome of this study was to identify differences in the results of the on-pump ROTEM examination between patients with and without endocarditis undergoing cardiac surgery. The secondary outcomes were the differences between the utilisation of red blood cells (pRBC), FC, PCC, FFP, and platelets. Further the differences in the results of the second ROTEM examination (conducted after heparin reversal), the coagulation management, and the postoperative blood loss between the two groups were examined.

### Measurements and Variables

The data extracted from the electronic health records (EHR) included demographic information, medical history, amount of blood products, and coagulation factor concentrates administered. The duration of extracorporeal circulation was noted on anaesthetic charts. ROTEM examinations were performed using a ROTEM Sigma (Werfen GmbH, Barcelona, Spain). The maintenance of the device, including regular quality controls, was conducted according to the manufacturer's recommendations. The results of the ROTEM assays (FIBTEM, EXTEM, HEPTEM, and INTEM) were recorded separately for each patient. The recorded ROTEM parameters included clotting time (CT), clot formation time (CFT), and clot firmness after 5 and 10 min (A5 and A10, respectively). In cases where the CFT approached infinity (ie, clot firmness did not reach 20 mm), the results were truncated at 1800s. Laboratory results before and after surgery were also documented. Blood loss was measured as hourly chest tube output over the first 24 h after surgery and documented by ICU nurses in patients’ records.

### Missing Data

All cases were excluded from the analysis if either CT or A5 was missing in the EXTEM or FIBTEM. Missing data from other recorded parameters were excluded from the analysis involving these parameters.

### Statistical Methods

All data were tested for missing data, outliers, and heteroscedasticity. Means with standard deviations or medians with the interquartile ranges and minimum-maximum ranges were reported to provide a representation of the dataset, including outliers. Categorical and binary data are presented as absolute frequencies with percentages. The propensity score was calculated for each patient using the variables relevant to the EuroSCORE II: New York Heart Association (NYHA) functional status, symptomatic angina pectoris, diabetes mellitus on insulin, age, sex, extracardiac arteriopathy, chronic pulmonary disease, reduced mobility, redo surgery, impaired kidney function, critical preoperative state, reduced left ventricular function, recent myocardial infarction, pulmonary hypertension, surgical urgency, and weight of the procedure (depending on number of operated valves and involvement of the thoracic aorta).^14^ Propensity score matching was conducted in a 1:1 ratio using the Matching package for R v. 4.10-14 (Sekhon JS) with a caliper of 0.2.^15^ Subsequently, the data were subjected to statistical analysis using JASP 0.19 (JASP Team, Amsterdam, The Netherlands) software.^16^ All data were checked for normal distribution using the Shapiro-Wilk test. Normally distributed data were compared using an unpaired t-test. Non-normally distributed data were compared using the Mann-Whitney U test. Statistical significance was set at *P* < .05.

## Results

### Participants and Descriptive Data

**Figure 1.**
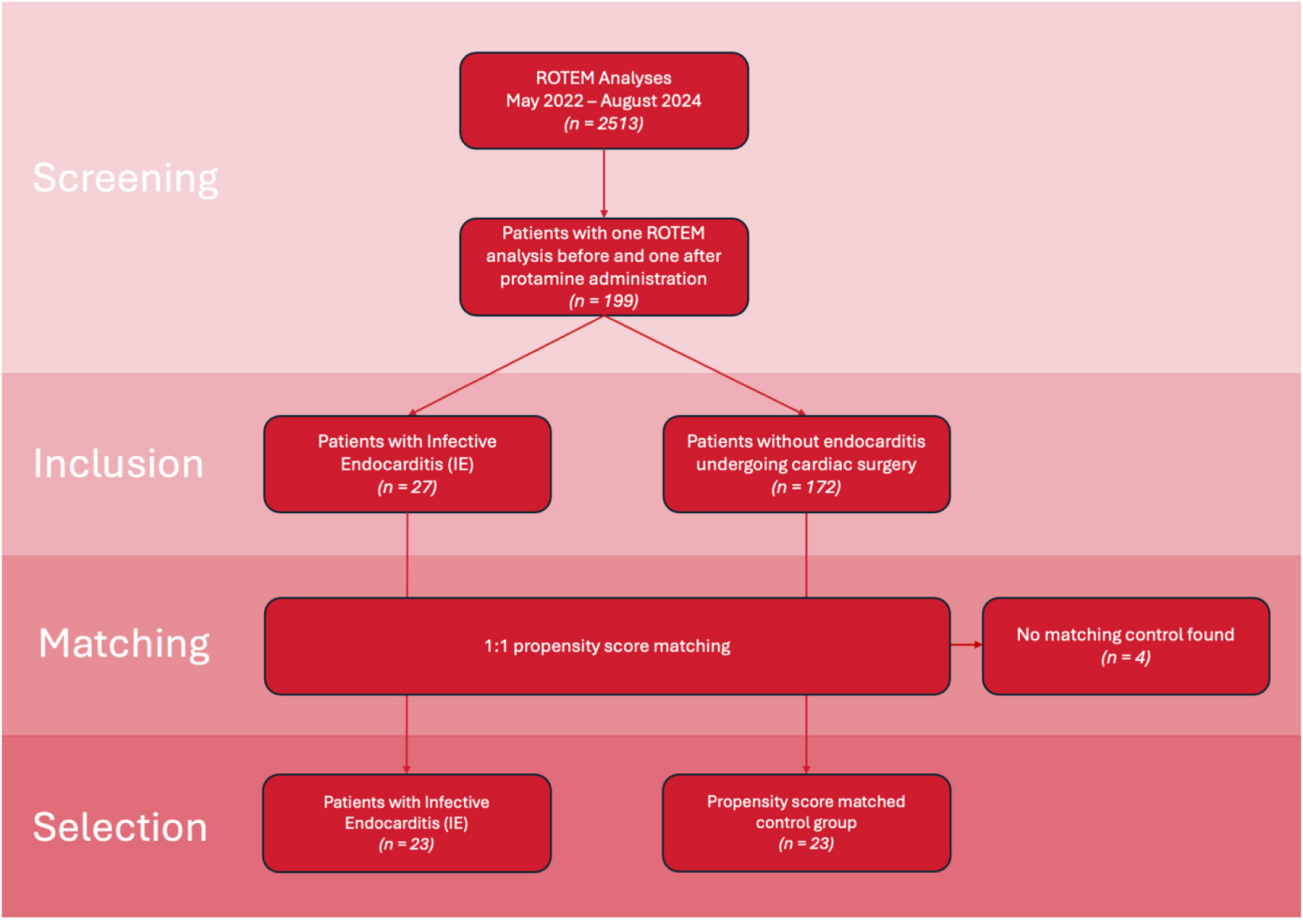
Flowchart of patient identification, matching and division into groups.

Screening of the ROTEM database yielded 199 patients with two ROTEM examinations: 27 eligible patients with IE and 172 patients without endocarditis. Patients with endocarditis were matched 1:1 to those without IE using the propensity score matching method described above. In four cases, no suitable match was found, and these patients were excluded from further analysis. 23 patients were analysed in each group: in the IE group, 20 male and 3 female patients aged 68 [IQR 64-72]; range 21-87, and in the non-IE group 15 male and 8 female patients aged 71 [IQR 61-74]; range 20-83. Preoperatively, haemoglobin level and thrombocytes count were significantly lower, INR higher and PTT longer in patients in the IE group. A flowchart of patient identification, matching, and division into groups is shown in Figure 1. The demographic and surgical data of the patients are presented in Table 1. Table 2 presents a summary of the results of the ROTEM test and the administered therapy.

**Table 1.** Demographic and surgical data of the included patients. Values are presented as mean (SD), median [IQR]; range, or number (proportion); BMI: Body Mass index, ASA: American Society of Anesthesiologists, CABG: Coronary Artery Bypass Graft, CPB: Cardio-Pulmonary Bypass.

Demographic Data	No IE Group	IE Group	*P*
Age; y	71 [61-74]; range 20-83	68 [64-72]; range 21-87	.652
Sex; male	15 [65%]	20 [87%]	.084
Height; cm	173 ± 11	176 ± 8	.323
Weight; kg	84 ± 22	86 ± 17	.397
BMI; kg ⋅ m^−2^	26.4 [24.8-29.4]; range 17.1-40.6	26.6 [23.2-31.5]; range 21.0-41.5	.959
ASA physical status			.565
1	0 (0%)	0 (0%)	
2	1 (4%)	0 (0%)	
3	3 (13%)	4 (17%)	
4	19 (83%)	19 (83%)	
EuroSCORE II; %	5.1 [2.9-20.4]; range 0.8-54.4	17.7 [6.8-29.2]; range 3.9-77.1	.016
Preoperative laboratory results			
Haemoglobin; mmol ⋅ l^−1^	7.8 ± 1.2	6.4 ± 1.1	<.001*
Thrombocytes; 10^3^ ⋅ l^−1^	200 [181-249]; range 70-384	176 [140-263]; range 35-744	<.001*
PTT; s	27 [25-29]; range 23-40	31 [29-35]; range 27-64	<.001*
INR	1.06 [1.0-1.25]; range 0.86-1.41	1.29 [1.15-1.46]; range 1.01-5.22	<.001*
CRP; mg ⋅ dl^−1^	3.5 [1.7-14.1]; range 0.0-101.0	48.7 [32.0-127.0]; range 3.8-414	<.001*
Surgical data			
Urgency; elective	11 (48%)	0 (0%)	<.001*
Urgency; urgent or emergent	12 (52%)	23 (100%)	<.001*
Type of procedure			.402
Isolated CABG	1 (4%)	0 (0%)	
Single non-CABG	9 (39%)	10 (43%)	
2 procedures	5 (22%)	9 (39%)	
3 procedures	1 (4%)	1 (4%)	
Surgery on thoracic aorta	3 (13%)	3 (13%)	
Time on CPB; min	94 ± 30	98 ± 50	.718

**Table 2.** Summary of the results of the ROTEM test and the administered therapy. Values are presented as mean ± SD, median [IQR]; range, or number (proportion). The INTEM results are not included in the on-pump test, as no clotting is anticipated in the presence of elevated heparin concentrations. The normal range values are given as in manufacturer documentation. ROTEM: Rotational Thromboelastometry, CT: Clotting Time, CFT: Clot Formation Time, A5 – Clot firmness after 5 min; A10 – Clot firmness After 10 min, PCC – 4-Factor Prothrombin Complex Concentrate, IBW – Ideal Body Weight, FFP – Fresh Frozen Plasma.

Assay	Parameter; Unit	Normal Range	No IE Group	IE Group	*P*
On-pump ROTEM					
FIBTEM	CT; s	46-84	97 [89-114]; range 72-268	134 [114-180]; range 87-287	<.001*
	CFT; s		1800 [1800-1800]; range 65-1800	345 [144-1744]; range 47-1800	.003*
	A5; mm	5-20	13 [11-15]; range 9-27	19 [16-23]; range 6-28	<.001*
	A10; mm	6-21	15 [12-16]; range 11-29	22 [18-25]; range 7-31	<.001*
EXTEM	CT; s	50-80	96 [92-123]; range 80-354	136 [115-191]; range 89-286	.001*
	CFT; s	46-149	66 [59-91]; range 36-239	58 [40-84]; range 28-416	.235
	A5; mm	32-52	43 ± 9	48 ± 12	.120
	A10; mm	43-63	49 [44-57]; range 36-54	50 [44-57]; range 17-65	.010*
HEPTEM	CT; s	160-211	271 [249-289]; range 212-380	303 [264-418]; range 216-868	.020*
	CFT; s	58-127	94 [70-131]; range 51-160	72 [60-108]; range 32-630	.088
	A5; mm	34-53	39 ± 8	45 ± 12	.052
	A10; mm	45-63	48 [42-56]; range 38-61	57 [47-60]; range 20-69	.129
Substitution between both tests					
Fibrinogen	g		0 [0-4]; range 0-8	0 [0-0]; range 0-6	.109
PCC	IU		2500 [2000-3000]; range 0-5000	3000 [3000-4000]; range 2000-5000	.014*
PCC	IU/kg IBW		40 ± 17	48 ± 13	.115
pRBC	unit		0 (0-2); range 0-5	0 (0-2); range 0-3	.332
Platelets	unit		0 [0-1]; range 0-2	0 [0-2]; range 0-3	.281
FFP	unit		0 [0-0]; range 0-4	0 [0-0]; range 0-4	1.000
ROTEM after heparin reversal					
FIBTEM	CT; s	46-84	76 [67-83]; range 55-177	84 [71-91]; range 64-112	.686
	CFT; s		1800 [482-1800]; range 22-1800	379 [103-1149]; range 59-1800	.018*
	A5; mm	5-20	17 ± 6	20 ± 4	.023*
	A10; mm	6-21	18 ± 6	21 ± 4	.106
EXTEM	CT; s	50-80	76 [72-83]; range 55-195	85 [74-90]; range 70-103	.124
	CFT; s	46-149	60 [47-74]; range 17-110	48 [36-55]; range 28-99	.010*
	A5; mm	32-52	47 ± 8	56 ± 7	<.001*
	A10; mm	43-63	55 ± 8	63 ± 6	.008*
INTEM	CT; s	161-204	260 [238-271]; range 189-370	273 [255-297]; range 184-477	.205
	CFT; s	62-130	79 ± 30	60 ± 20	.016*
	A5; mm	33-52	42 ± 8	51 ± 7	<.001*
	A10; mm	43-62	52 ± 8	59 ± 7	.016*
HEPTEM	CT; s	160-211	241 [238-265]; range 186-346	282 [244-310]; range 172-490	.119
	CFT; s	58-127	87 ± 33	64 ± 20	.006*
	A5; mm	34-53	40 ± 7	48 ± 7	.002*
	A10; mm	45-63	51 ± 8	57 ± 7	.030*

### Main Results

**Figure 2.**
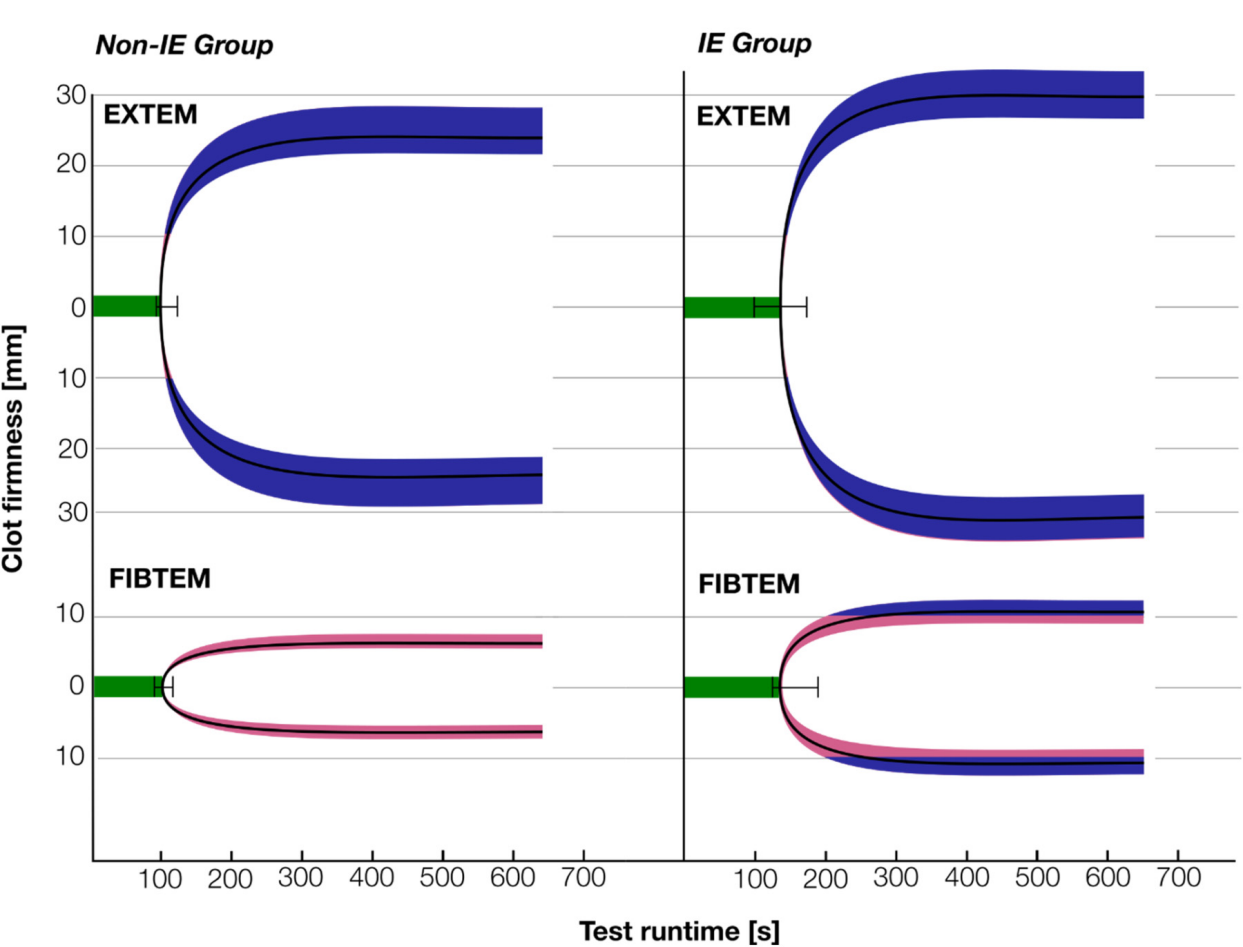
Illustration of the ROTEM results during the end-phase of the cardiopulmonary bypass. Green lines represent the mean clotting times (CT), with IQR represented by whiskers. Black lines represent the mean clot firmness at a given runtime, with IQR represented by the pink (for clot firmness <20 mm) and blue (for clot firmness >20 mm) areas; IE: infective endocarditis.

**Figure 3.**
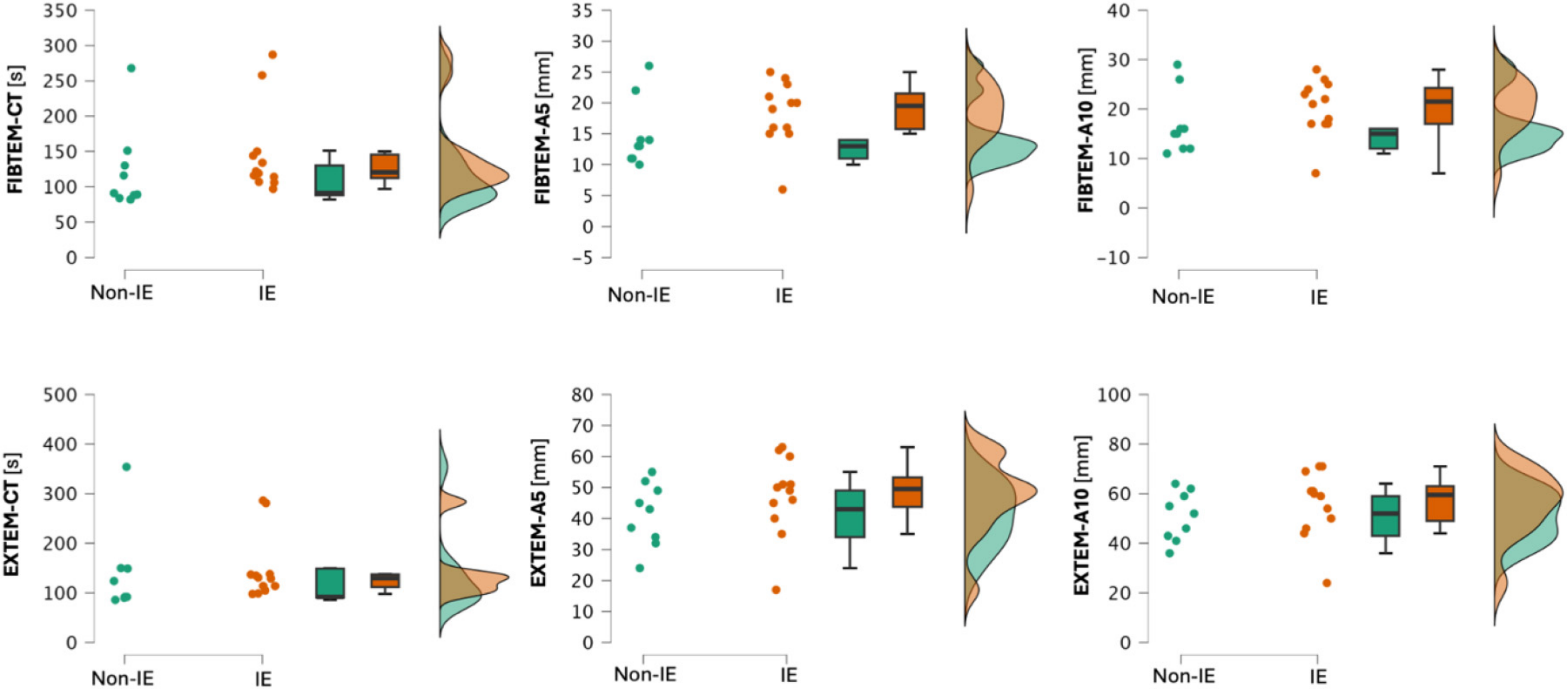
Raincloud plots presenting the results of the main statistical analysis. The clouds of points represent single data points in the dataset, box plots: horizontal line represents the median, box represents the IQR, and whiskers minimum and maximum values. One-sided violin plot depicts the distribution of the data.

Statistically significant differences were identified in the on-pump FIBTEM between the IE and control groups with regard to CT (longer in IE group, *P* < .001), A5, and A10 (both higher in IE group with *P* < .001). In the on-pump EXTEM, the differences in CT and A10 were found to be statistically significant (*P* = .001 and *P* = .010, respectively), while the difference in A5 did not reach statistical significance (*P* = .120). In the IE group, 20 out of 23 patients (87%) had coagulopathy, defined as CT > 100 s in EXTEM, compared to 10 out of 23 patients (43%) in the non-IE group. The numerical results of these analyses are shown in Table 2 and Figures 2 and 3.

### Other Analyses

#### Transfusions of Blood Products and Coagulation Factors

**Figure 4.**
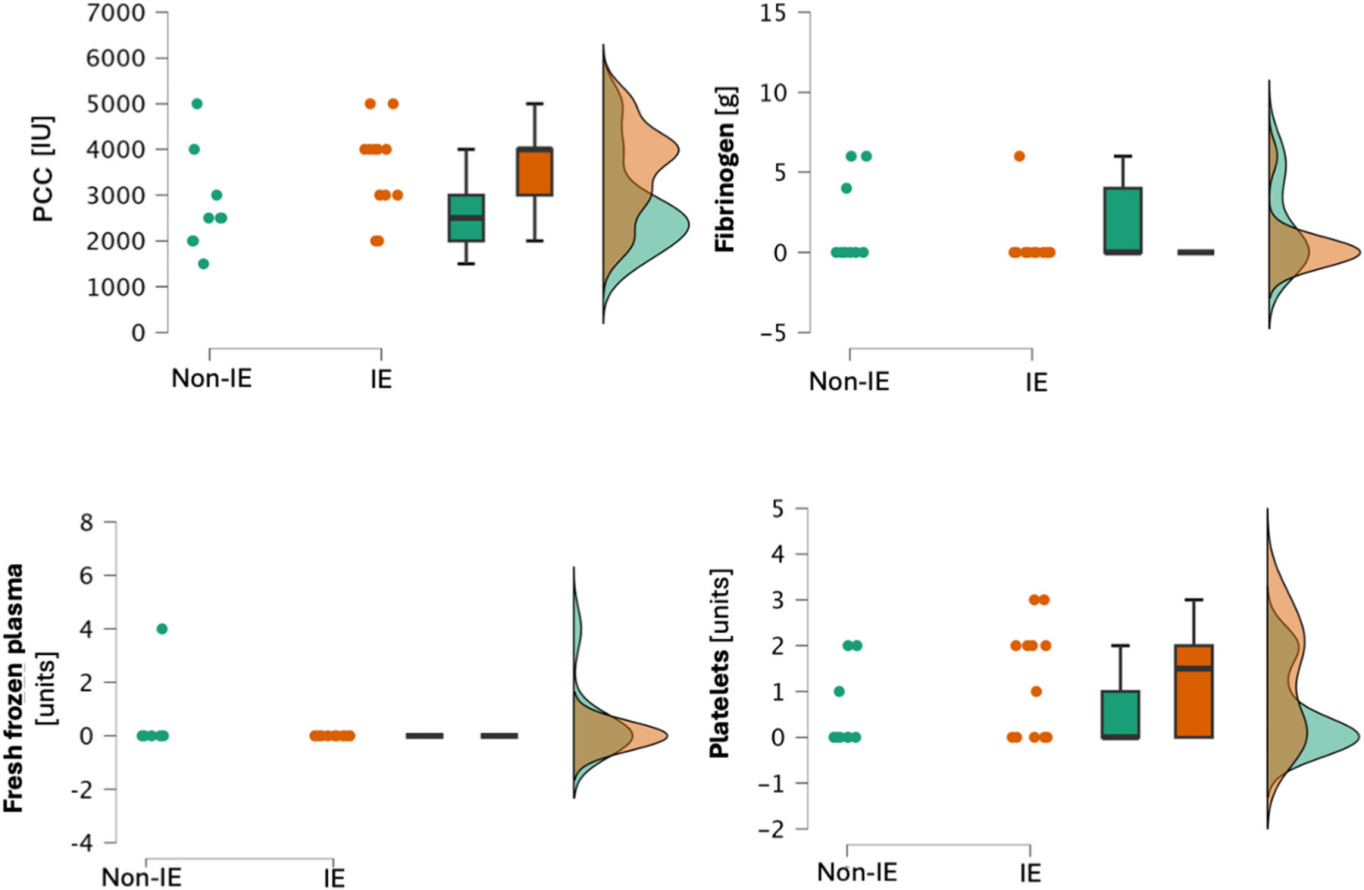
Raincloud plots presenting the analysis of transfused blood products and clotting factors. The clouds of points represent single data points in the dataset, box plots: horizontal line represents the median, box represents the IQR, and whiskers minimum and maximum values. One-sided violin plot depicts the distribution of the data.

We found no statistically significant differences in the amount of intraoperatively transfused red blood cells, fresh frozen plasma, or platelets. The amount of fibrinogen concentrate given also failed to reach the statistical significance threshold. The absolute amount of PCC (median 3000 units in the IE group vs 2500 units in the non-IE group, *P* = .014) but not PCC per kilogram of ideal body weight was significantly higher in the IE group than in the non-IE group. The results of this analysis are shown in Table 2 and Figure 4.

Notably, the total (i.e. administered until discharge from the hospital) number of transfused units of red blood cells (median 5 units in the IE group vs 1 unit in the non-IE group, *P* = .006), fresh frozen plasma (median 5 units vs 1 unit, *P* = .038), but not platelets (median 2 units vs 0 units, *P* = .089) was significantly higher in the IE group than in the non-IE group.

#### ROTEM Results After Supplementation

After the administration of protamine and supplementation of clotting factors and/or platelets and fresh frozen plasma, there were no significant differences in clotting time in either ROTEM assay (FIBTEM CT *P* = .686; EXTEM CT *P* = .124, INTEM CT *P* = .205). However, A5 remained significantly different in all assays (FIBTEM *P* = .023; EXTEM *P* < .001; INTEM *P* < .001) and A10 in EXTEM (*P* = .008) and INTEM (*P* < .001), but not in FIBTEM (*P* = .106). The results of this analysis are shown in Table 2.

Noteworthy, both the absolute change in CT in EXTEM (median, [IQR], range: −57 s, [−97 to −41]; −215 to −2 vs −25 s, [−41 to −16]; −159 to −6, *P* = .003) and the change in CT per unit PCC per kg ideal body weight (−1.1 s ⋅ unit^−1^ ⋅ kg^−1^, [−2.2 to −0.8]; −4.2 to −0.05 versus −0.7 s ⋅ unit^−1^ ⋅ kg^−1^, [−1.0 to −0.5]; −2.4 to −0.2, *P* = .022) were significantly higher in the IE group than in the non-IE Group.

#### Postoperative Blood Loss

When comparing chest tube output in the first 24 h postoperatively, there were no differences between the IE group and the No IE group (725 ml, [400-1044]; 150-6400 vs 525 ml, [363-900]; 200-2300, *P* = .565).

## Discussion

### Key Results

Our findings indicate that patients with IE undergoing cardiac surgery exhibit prolonged clotting times and augmented clot firmness (as represented by A5 and A10 values) when compared to similar patients without IE. Based on our on-pump ROTEM analysis, lower FC and higher PCC doses were necessary to restore optimal coagulation in this cohort. It is noteworthy that after supplementation with clotting factors, the improvement in clotting time observed in the IE Group was more pronounced than in the non-IE Group, with a greater reduction in clotting time per IU of PCC per kilogram of ideal body weight. The number of transfused platelets and fresh frozen plasma did not differ significantly between the groups. Following heparin reversal, the clot firmness in the IE group remained greater, but there were no differences in clotting time.

### Interpretation

The findings of our study demonstrate that patients with IE exhibit a distinctive pattern of hypercoagulability, as evidenced by clot firmness accompanied by indications of consumption coagulopathy, as indicated by prolonged clotting time. Furthermore, to the best of our knowledge, this study represents the first comparison of the clotting factor doses administered to patients with and without infective endocarditis, and the impact of these treatments on subsequent rotational thromboelastometry results.

However, other researchers have analysed coagulation in patients with IE. Koltsova et al reported general platelet pre-activation and hypercoagulability. Some patients in their study exhibited a shift and their INR and thromboelastography results were similar to those observed in our study.^11^ These findings suggest that the surgical population aligns with the described subpopulation, which shifts to consumption coagulopathy.^17^

A study by Breel et al compared the ROTEM profiles of surgical patients with and without IE before (after induction of anesthesia), during (after aortic declamping), and after surgery. Notably, the defined time points differed from those used in the present study. In line with our findings, they preoperatively found a longer CT and greater A5 in the IE group.^18^ However, the differences in their findings were lower than those in our study, possibly suggesting the crucial role of CPB in the further deterioration of coagulation in this patient population. Furthermore, the authors of the referenced study indicated that 24 of 31 patients with IE received PCC. While the precise timing of PCC administration was not specified in their paper, we hypothesize that it occurred after intraoperative ROTEM. In our study, all IE patients received PCC, and the second ROTEM test was conducted after the administration of PCC and FC. The discrepancies between the results could be explained by differences in cardiopulmonary bypass procedures, although a direct comparison is not possible without further details on the latter. Despite the discrepancies in the ROTEM results observed in our investigations, the IE patients enrolled in both studies received a greater dosage of PCC and a higher amount of RBC, FFP, and platelets. In accordance with the findings of the aforementioned study, our observations revealed no difference in the FC dosage between the groups.

A micropathological triad of thrombosis has been proposed as a potential framework for understanding the underlying mechanisms involved. This phenomenon is characterised by a reduction in coagulation time in non-activated tests, which is indicative of the expression of tissue factors in circulating cells and microparticles. Additionally, it is accompanied by hypofibrinolysis and hypercoagulability, as defined by an increase in overall clot firmness and fibrinogen contribution to clot firmness.^19–23^ Analysis of fibrinolysis parameters was not conducted in the present study because of the limited duration of the tests. Furthermore, our institutional protocol mandates the administration of one gram of tranexamic acid to all patients undergoing cardiac surgery following the anaesthesia induction. This is likely to affect the degree of fibrinolysis observed in our patients, thereby precluding us from drawing meaningful conclusions regarding preoperative fibrinolysis. No patient exhibited subnormal coagulation times, and no cases of thrombotic complications were observed following surgery.

Further research is required to ascertain whether a strategy of tolerating certain degrees of prolonged CT is more effective in reducing the risk of thrombosis while maintaining safety regarding bleeding risk. It would also be beneficial to gain a deeper understanding of the potential benefits and risks associated with the use of tranexamic acid in patients with hypercoagulability, as evidenced by increased clot firmness in the FIBTEM and EXTEM tests.

### Limitations

We acknowledge the limitations of this study. First, owing to the retrospective nature of the study, there was no fixed duration for the viscoelastic tests. This resulted in some missing data, as clinicians tend to terminate the tests once they have obtained the required clinical information. Specifically, we were unable to compare the maximal clot firmness and lysis parameters owing to the insufficient runtime of all tests. Second, the sample size was relatively small, which precluded our ability to assess the incidence of postoperative complications such as thrombosis. It is also possible that the study population may have been subjected to a selection bias, as the decision to conduct a second ROTEM test was made by the treating clinicians based on clinical judgement and personal preferences. In some cases, this decision may have been influenced by the results of the first ROTEM test, in case they were within the normal range.

As in most cases of cardiac surgery, the coagulation status is clinically relevant only after weaning from cardiopulmonary bypass, the attention of cardiac surgeons and cardiac anaesthetists is focused on the unique challenges of coagulation management at this time. Consequently, no baseline ROTEM results were obtained for this retrospective study, since such measurements are not part of our standard clinical practice.

Another limitation is that all patients received tranexamic acid. In one pilot study, TXA was shown to cause paradoxical changes in coagulation status, resulting in prolonged CT at sternotomy (though not at the end of cardiopulmonary bypass) and reduced clot firmness.^24^ Nevertheless, the administration of TXA has become widespread in cardiac surgery, reflecting current clinical practice. As both groups of patients received TXA, it was unlikely to influence the differences between them.

## Conclusion

Patients with infective endocarditis undergoing on-pump cardiac surgery exhibit distinctive viscoelastic profiles characterized by prolonged clotting time and elevated maximum clot firmness. They are more prone to transfusion of blood products and prothrombin complex concentrate supplementation. Further research is necessary to establish the optimal target values in ROTEM tests with the aim of minimizing bleeding risk while avoiding an increase in thrombotic risk.

## List of Abbreviations

A10: Clot firmness after 10 min
A5: Clot firmness after 5 min
ACT: Activated coagulation time
CFT: Clot formation time
CT: Coagulation time
EHR: Electronic Health Records
FFP: Fresh frozen plasma
FC: (Human) fibrinogen concentrate
IE: Infective endocarditis
NYHA: New York Heart Association
PCC: 4-factor prothrombin complex concentrate
pRBC: packed Red Blood Cells
ROTEM: Rotational Thromboelastometry
